# Social Return on Investment (SROI) methodology to account for value for money of public health interventions: a systematic review

**DOI:** 10.1186/s12889-015-1935-7

**Published:** 2015-06-24

**Authors:** Aduragbemi Oluwabusayo Banke-Thomas, Barbara Madaj, Ameh Charles, Nynke van den Broek

**Affiliations:** Centre for Maternal and Newborn Health, Liverpool School of Tropical Medicine, Liverpool, L3 5QA UK

**Keywords:** Value for money, Health economics, SROI, Social impact, Impact evaluation, Evaluation research, Health inequalities, Blended value accounting, Triple bottom line, Public health

## Abstract

**Background:**

Increased scarcity of public resources has led to a concomitant drive to account for value-for-money of interventions. Traditionally, cost-effectiveness, cost-utility and cost-benefit analyses have been used to assess value-for-money of public health interventions. The social return on investment (SROI) methodology has capacity to measure broader socio-economic outcomes, analysing and computing views of multiple stakeholders in a singular monetary ratio. This review provides an overview of SROI application in public health, explores lessons learnt from previous studies and makes recommendations for future SROI application in public health.

**Methods:**

A systematic review of peer-reviewed and grey literature to identify SROI studies published between January 1996 and December 2014 was conducted. All articles describing conduct of public health SROI studies and which reported a SROI ratio were included. An existing 12-point framework was used to assess study quality. Data were extracted using pre-developed codes: SROI type, type of commissioning organisation, study country, public health area in which SROI was conducted, stakeholders included in study, discount rate used, SROI ratio obtained, time horizon of analysis and reported lessons learnt.

**Results:**

40 SROI studies, of varying quality, including 33 from high-income countries and 7 from low middle-income countries, met the inclusion criteria. SROI application increased since its first use in 2005 until 2011, declining afterwards. SROI has been applied across different public health areas including health promotion (12 studies), mental health (11), sexual and reproductive health (6), child health (4), nutrition (3), healthcare management (2), health education and environmental health (1 each). Qualitative and quantitative methods have been used to gather information for public health SROI studies. However, there remains a lack of consensus on who to include as beneficiaries, how to account for counterfactual and appropriate study-time horizon.

Reported SROI ratios vary widely (1.1:1 to 65:1).

**Conclusions:**

SROI can be applied across healthcare settings. Best practices such as analysis involving only beneficiaries (not all stakeholders), providing justification for discount rates used in models, using purchasing power parity equivalents for monetary valuations and incorporating objective designs such as case–control or before-and-after designs for accounting for outcomes will improve robustness of public health SROI studies.

**Electronic supplementary material:**

The online version of this article (doi:10.1186/s12889-015-1935-7) contains supplementary material, which is available to authorized users.

## Background

Recognising the need to institute a culture of accountability, funders of public healt interventions and national governments are demanding “value for money” (VfM) of interventions, to ensure both economic and social efficiency and better allocation of resources for the wider good of the people [1–3]. It is important and timely to review assessment frameworks that attempt to demonstrate this value and their applicability in the public health area.

Traditionally, frameworks such as cost-effectiveness analysis (CEA), cost-utility analysis (CUA) and cost-benefit analysis (CBA) have been used [4]. However, in recent times, social return on investment (SROI) methodology has been promoted as a more ‘holistic’ approach to demonstrating VfM [5–7]. A comparison of the different approaches is provided in Table 1.

**Table 1:** Comparison of SROI with traditional economic evaluation frameworks

Cost-Effectiveness Analysis (CEA)	Cost-Utility Analysis (CUA) Sub-type of CEA	Cost-Benefit Analysis (CBA)	Social Return on Investment (SROI)
Main objective			
To compare costs and impact of alternatives within the same domain	To compare costs and impact of alternatives within the same domain	To assess if an intervention is worth the investment.	To assess if an intervention is worth the investment.
Costs			
Monetary value	Monetary value	Monetary value	Monetary value
Benefits			
Benefits linked to health improvements.	Benefits linked to health improvements.	Captures health and non-health impacts.	Captures health and non-health impacts, underpinned by the “triple bottom line” approach (social, economic and environmental). In addition, seeks to account for and value potential negative effect of interventions.
Reported as natural units E.g. lives saved or cases averted	Reported as Quality Adjusted Life Years (QALYs) gained/ Disability Adjusted Life Years (DALYs) averted/ Healthy life-years gained	Reported as monetary value or welfare benefit	Reported as monetary value or welfare benefit
		Lists benefits that cannot be easily monetised and explains why they cannot be monetised	Uses financial proxies to estimate monetary value of benefits that cannot be easily monetised
Level of application			
Intervention level	Intervention level	Usually intervention level	Intervention, project, programme, policy or organisation level
Timeline of analysis			
Retrospective or Prospective	Retrospective or Prospective	Retrospective or Prospective	Retrospective or Prospective
Discounting of future value			
Yes	Yes	Yes	Yes
Stakeholder engagement			
No	No	No	Yes
Theory of change			
No	No	No	Yes
Main output of analysis			
Incremental Cost-Effectiveness Ratio (ICER)	Incremental Cost-Effectiveness Ratio (ICER)	Benefit-Cost Ratio (BCR)	Social Return on Investment Ratio
		Economic Internal Rate of Return (EIRR)	Net Present Value (NPV)
		Net Present Value (NPV)	Payback period
		Break-even point	
Interpretation of main output of analysis			
Intervention with higher cost-effectiveness ratio is better	Intervention with higher cost-effectiveness ratio is better	BCR > 1 is worthwhile investment	SROI ratio > 1 is worthwhile investment
Relevance			
Priority setting and resource allocation	Priority setting and resource allocation	Priority setting and resource allocation	Priority setting
			Resource allocation
			Stakeholder relationship building,
			Accountability framework, Management tool

In the most recent SROI methodology guidance, SROI is defined as “a framework for measuring and accounting for the much broader concept of value. It seeks to reduce inequality and environmental degradation and improve wellbeing by incorporating social, environmental and economic costs and benefits” [8]. SROI is a process for understanding, measuring, and reporting the social, economic and environmental value created by an intervention, programme, policy or organisation [9]. SROI can retrospectively measure outcomes that have already occurred (evaluative-type) or can prospectively predict how much value will be generated if the intervention meets its intended outcomes (forecast-type) [8]. Data collection and subsequent analyses allow calculation of a benefits-to-costs ratio [8]. For example, a ratio of 4:1 indicates that an investment of £1 delivers £4 of social value.

**Fig. 1:**
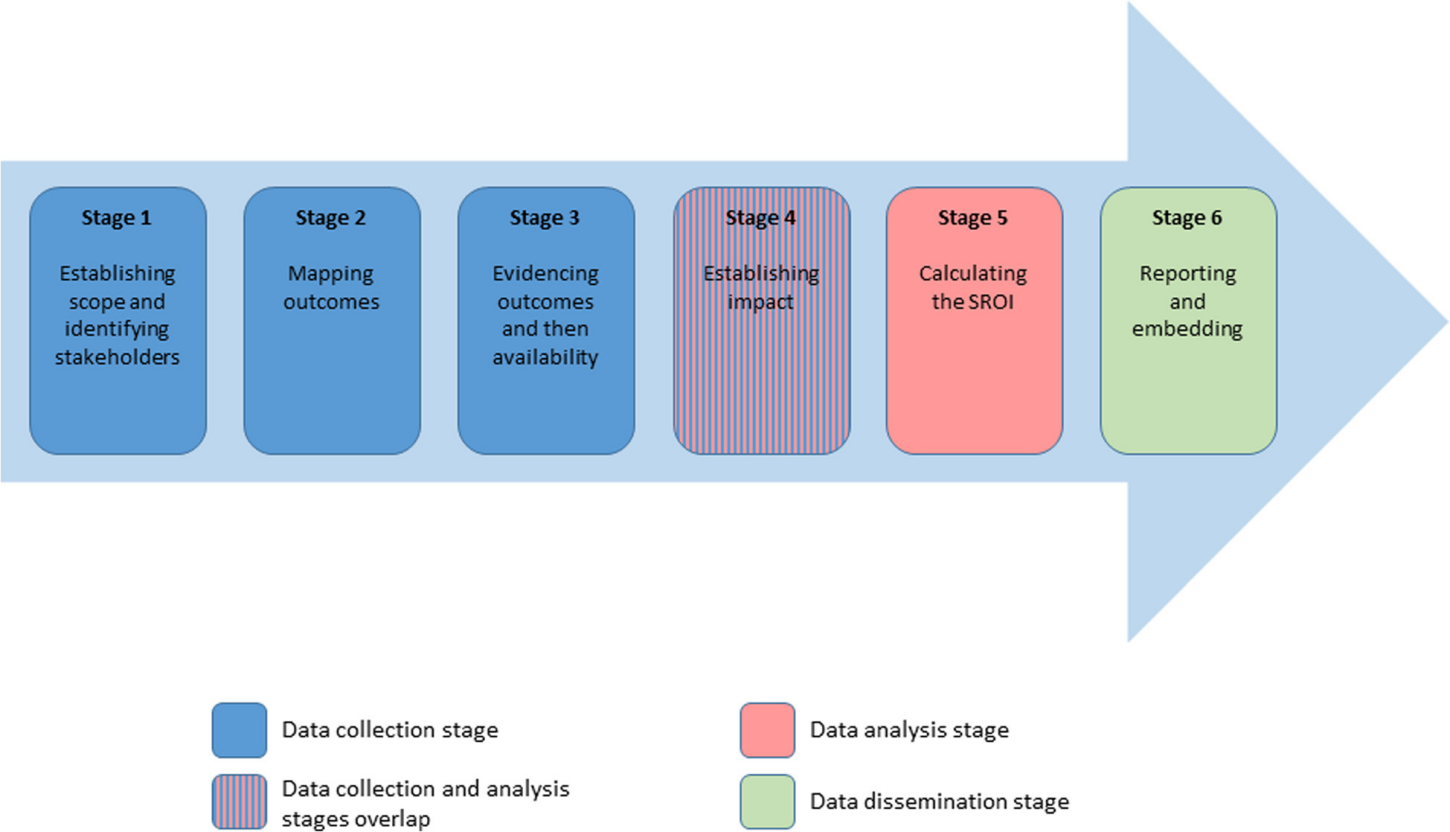
Stages of the SROI process

The SROI framework was first developed by the Roberts Enterprise Development Fund (REDF) in 1996 [10], after which there has been a gradual revision of the original methodology [11]. These revisions have led to an integration of REDF‘s original SROI methodology (a social impact measurement tool) with principles and processes normally used in economic evaluations and financial return on investment to build a framework capable of capturing the wider impact of interventions (social, economic and environmental) [12]. This concept is widely referred to as the “triple bottom line” [13], which is in itself underpinned by the “blended value accounting” theory [14]. Based on the most recent guideline [8], the conduct of a SROI study requires progression through six stages [Fig. 1].

Previous narrative reviews have appraised the SROI methodology, putting forward its strengths (including capacity to generate a singular ratio that captures both positive and negative outcomes, provision of platform for meaningful engagement of multiple stakeholders and its representation of stakeholder benefits in ways that are unique to the stakeholders themselves) and weaknesses (difficulty of attaching financial values to “soft outcomes” and establishing what would have happened without the intervention (the counterfactual) as well as poor comparability of SROI ratios across interventions) [6, 7, 10, 15]. Two other reviews have compared SROI with social impact measurement tools such as Social Accounting and Auditing (SAA) and the Global Reporting Initiative (GRI) highlighting that SROI is the only methodology that captures change across the whole spectrum of the theory of change (input – impact) and provides a monetised ratio [11, 16]. When compared to traditional economic evaluation tools (CEA, CUA, CBA) [17, 18], SROI has been described as an extension of the CBA to incorporate in addition the broader socio-economic and environmental outcomes [6, 7, 10, 11, 15]. SROI is able to achieve this through its use of financial proxies, allowing complex outcomes such as ‘reduced stigma for people living with HIV/AIDS’ to be accounted for [19]. Another review compared guidance on these methodologies for economic evaluation of public health interventions including SROI, suggesting that the techniques proposed for SROI guidance relate well to public health [20]. Furthermore, there is a non-peer reviewed narrative review that explores the application of the SROI methodology across different sectors [21]. However, there is no previous systematic review that focuses on the application of SROI specifically in public health.

We, therefore, conducted a systematic review of the peer-reviewed and grey literature to identify and assess studies in which the SROI methodology has been applied in public health, explore lessons learnt based on previous applications and make recommendations for future SROI application in public health.

## Methods

We followed the PRISMA approach [22] to reporting the findings of this systematic review of SROI application of public health interventions [Additional file 1].

### Search strategy

The preliminary search terms used were “social return on investment” and “SROI”. After an initial review of identified studies, the search terms were expanded to include: “blended value accounting”, “return on investment”, “ROI”, “economic return on investment”, “social rates of return on investment”, "social value" and “social impact". However, when used, not all terms were found to be sensitive for “social return on investment”. Following this exploration, the decision was made to use the search terms “social return on investment”, “SROI” and “blended value accounting” which were combined with “health” OR “public health” within peer reviewed databases (PubMed, Scopus and ProQuest).

For grey literature, SROI studies were identified via review of titles, abstracts or executive summaries or full text of articles found through web search (Google Scholar) or from SROI focused databases (SROI Network and new economics foundation (nef)).

For both peer reviewed and grey literature sources, we searched for articles published from January 1996 and December 2014. This time frame was chosen because the first recorded SROI report was published in 1996.

We hand-searched the content pages of journal issues and reports and checked reference lists of identified articles to identify additional studies. Direct emails were sent to practitioners, whose contact details were available in executive summaries or websites that made reference to conduct of a public health SROI study, to request reports of these SROI studies. In addition, a public request was made to relevant SROI interest online groups to ensure that all public health SROI studies were potentially captured.

Two researchers independently conducted the search and reviewed all retrieved records. Agreement was reached regarding the final eligibility of articles based on the set inclusion and exclusion criteria. Opinion of a third reviewer was requested when consensus was not reached.

### Inclusion and exclusion criteria

Public Health SROI articles from both peer-reviewed and grey literature sources, published in English, which described actual conduct of the study and included a SROI ratio, from 1996 onward were included.

Articles that measured social impact using other approaches than SROI, reviews, commentaries and editorials as well as articles that only referred to SROI without any detail on actual conduct of a SROI were excluded.

### Data extraction and synthesis

A pre-developed summary table was used to capture year of publication, type of SROI study, country of organisation conducting or commissioning the SROI study, type of commissioning organisation, country where study was conducted, public health area in which SROI was conducted, stakeholders included in study, stakeholder classification, discount rate used in the study, SROI ratio obtained, time horizon of analysis (Intervention-Measurement) and reported lessons learnt. Missing or unclear information was obtained by contacting the author(s) of the SROI article directly, wherever possible.

Thematic summaries were used to configure and compare information obtained. Findings retrieved from the studies were summarised to map patterns in the application of the SROI methodology in public health. To analyse information on lessons learnt with regard to limitations and strengths of the methodology, the deductive approach of framework synthesis [23] was used. Findings are presented as emerging themes.

### Definitions

For the purpose of this review, a stakeholder was defined as “a person who is affected by the work of an organisation or has contributed to the work” [24]. Borrowing from previous stakeholder classification frameworks [25–27], we defined the different types of stakeholders as:

i. Beneficiaries: users, those who experience the outcomes of an intervention.
ii. Implementers: includes project managers, suppliers and subcontractors.
iii. Promoters: those who provide support and a conducive environment for implementation of the intervention.
iv. Funders: those who finance the project.

### Quality assessment

A 12-point quality assessment framework, developed by Krlev et al., [21] [Table 2] was used to assess quality of included SROI studies. This framework proposed 5 quality dimensions:

I. Transparency about why SROI was chosen
II. Documentation of the analysis
III. Study design (approximation of counterfactual)
IV. Precision of the analysis and
V. Reflection of the results.

**Table 2:** Krlev et al’s 12-point quality assessment framework

Dimension number	Dimension	Criterion	Description of criterion
I	Transparency about why SROI was chosen	1	Linked to context discussion?
II	Documentation of the analysis	2	Analysis well documented?
		3	Impact map used?
III	Study design (approximation of ‘dead-weight’)	4	Control group setup applied?
		5	Ex ante - ex post observations performed?
IV	Precision of the analysis	6	Indicators valid & comprehensive?
		7	Proxies valid & comprehensive?
		8	Social effects captured? (Qualitatively)
		9	Social effects captured? (Quantitatively)
V	Reflection of the results	10	Limitations discussed?
		11	SROI ratio interpreted?
		12	Sensitivity analysis performed?

An award of 1 point was given to each criterion that was adjudged “present” and 0 if the item was “missing” or “could not be ascertained”. We used the 70 % benchmark, which Krlev et al. describe as a “good score”, classifying papers into high quality, if the study scored ≥ 70 % and low quality, if the study scored < 70 %.

## Results

**Fig. 2:**
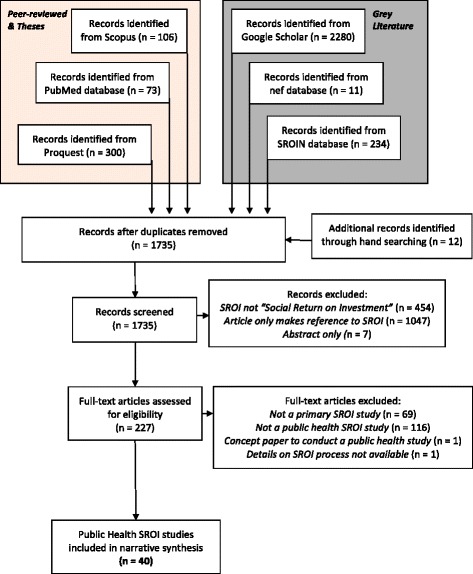
PRISMA flowchart summarising the search process

A total of 40 SROI studies were identified; 82.5 % were retrieved from grey literature, while 10 % were from peer-reviewed journals. The remaining 7.5 % were from online repositories of theses (Bachelors, Masters or Doctorate) [Fig. 2].

Of the 40 included SROI studies, between 83 and 100 % were awarded “1 point” for the presence of the specific quality criteria across three quality dimensions: transparency about why SROI was chosen, documentation of the analysis and precision of the analysis. However, with the remaining two quality dimensions (study design [approximation of dead weight] and reflections of the result), percentages of studies with presence of specific criteria ranged from 18 % to 48 %. The majority of studies did meet the criteria ‘SROI ratio interpreted’ (100 %) and ‘sensitivity analysis performed’ (80 %) [Additional file 2].

For the criterion ‘study design (approximation of dead weight)’, 8 studies used control groups [19, 28–34] (to establish what would have happened without the intervention, while another 8 studies used a before-and-after study design [35–42]. The remainder of the studies based the estimation on what would have happened without the intervention on assumptions or on opinions of the stakeholders that were engaged for the purpose of the study [Additional file 2].

For the criterion ‘reflection of the result’, 19 studies discussed the limitations of the study. Eight studies did not conduct sensitivity analysis to test robustness of assumptions used in the conduct of the study [29, 35, 40, 43–47]. However, all studies calculated and interpreted the resultant SROI ratio [Additional file 2].

Out of a maximum of 12 that could be achieved, quality scores ranged from 3 to 11 (median 9). Twelve public health SROI studies were considered to be of low quality [28, 31, 37, 40, 41, 43, 45, 46, 48–51], while the remaining 28 were of high quality [19, 29, 30, 32–36, 38, 39, 42, 44, 47, 52–66]

### Distribution of SROI application in public health

**Fig. 3:**
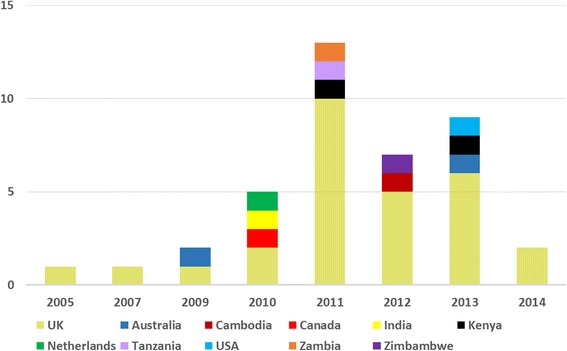
Number of studies published by year in countries where SROI has been applied

The application of SROI in public health steeply increased since its first use in the UK in 2005 [63] until 2011, after which there has been a decline [Fig. 3].

28 SROI studies in public health were conducted in the UK [30, 32, 33, 37–42, 44–48, 50, 52–64]. Other countries in which the SROI methodology has been applied in public health are Australia [49, 65], Kenya [29, 34], Cambodia [51], Canada [35], India [36], Netherlands [28], Tanzania [43], United States of America [31], Zambia [19] and Zimbabwe [66] [Fig. 3].

## Characteristics of included studies

Different organisations and individuals within the public, profit and non-profit sectors have used SROI to evaluate a range of interventions in different areas of public health [Additional file 3].

Across public health areas, health promotion (12) [28, 30, 33, 39, 42, 46, 50, 60–62, 65, 66] and mental health (11) [32, 37, 45, 47, 49, 52, 53, 55, 57, 58, 63] are areas in which the SROI methodology has been most applied. The methodology has also been applied in sexual and reproductive health (SRH) (6) [19, 34, 38, 51, 54, 56], child health (4) [31, 36, 41, 43], nutrition (3) [33, 44, 49] and to a smaller degree in health care management (2) [35, 64], environmental health (1) [29] and health education (1) [40] [Table 3].

**Table 3:** Findings from systematic review of SROI application in Public Health

Study characteristics	Number of studies	% of total
SROI type		
Evaluative type	26	65.00
Forecast type	14	35.00
Area of Public Health		
Child Health	4	10.00
Environmental Health	1	2.50
Health Care Management	2	5.00
Health Education	1	2.50
Health Promotion	12	30.00
Mental Health	11	27.50
Nutrition	3	7.50
Sexual Reproductive Health	6	15.00
Stakeholders included		
Only beneficiaries	21	52.50
Beneficiaries and implementers	3	7.50
Beneficiaries and promoters	2	5.00
Beneficiaries, implementers and promoters	3	7.50
All stakeholders	11	27.50
Data Source		
Qualitative alone	3	7.50
Qualitative + primary	8	20.50
Qualitative + secondary	9	22.50
Qualitative + primary + secondary	15	37.50
Quantitative (primary) alone	1	2.50
Quantitative (secondary) alone	3	7.50
Quantitative (primary + secondary)	1	2.50

26 of the retrieved SROI studies were evaluative-type [29–36, 40, 42–46, 49–55, 60, 61, 63–65], while the other 14 were forecast-type SROI studies [28, 37–41, 47, 48, 56–59, 62, 66] [Table 3].

### Data collection for SROI

The SROI methodology is firmly based on retrieving perspectives of stakeholders [8] (SROI Stage 1) [Fig. 1]. Most SROI studies (29) identified all stakeholders before choosing which group of stakeholders to include in the SROI analysis. The remaining 11 studies only considered beneficiaries [Additional file 3].

A breakdown of stakeholder inclusion for final analysis following initial consideration revealed that 12 studies included all stakeholders (beneficiaries, promoters, implementers and funders) [19, 28, 29, 39, 41, 53, 59, 60, 62–64, 66]; 3 studies included beneficiaries and implementers [32, 46, 54]; 2 studies included beneficiaries and promoters [34, 37]; another 3 studies included beneficiaries, promoters and implementers [44, 48, 50]. Twenty-one studies included only beneficiaries of the intervention [28, 30, 31, 33, 35, 36, 38, 40, 42, 43, 45, 47, 49, 51, 52, 55–58, 61, 65] [Table 3].

From the included stakeholders, information such as inputs required for the intervention (costs, time, etc.), perceived changes experienced by the stakeholder as a result of the intervention, outcomes benefited or otherwise from the intervention, duration of the outcome, relative importance or prioritisation of these outcomes, changes likely to have occurred in the absence of the intervention and other factors contributing to the changes identified were gathered to build the SROI impact map [30, 32, 35, 60].

To gather this information, eight studies used a mix of qualitative and primary quantitative data [25, 36, 44, 53, 54, 61, 62, 66], 15 studies used a combination of qualitative, primary quantitative and secondary (existing) quantitative data [19, 30, 32, 33, 38, 39, 42, 47, 52, 55, 57–60, 65], 9 studies used a combination of qualitative and secondary quantitative data [29, 39, 41, 43, 45, 46, 56, 63, 64], 3 studies used only qualitative data [37, 48, 51] and 3 studies used secondary quantitative data alone [28, 31, 49]. One study used primary quantitative data alone and another combined primary and secondary quantitative data [40, 50] [Table 3].

### Calculation of SROI ratio

Among the 26 evaluative public health SROI studies, the median duration between implementation of the intervention and assessment of SROI was 1 year and 11 months (range of 4 months to 5 years). Meanwhile, forecast public health SROI studies had a median duration of 9 years and 5 months (range of 1 to 30 years).

Discount rates, used to account for future value of costs and benefits [67], varied depending on specific country recommendation (for example, 3.5 % is recommended in the UK) and this was the justification provided in all studies for the choice of the discount rate used in the model [Additional file 3].

SROI ratios varied across the different public health areas, with the highest ratio of 65.0:1 reported in a study in child health and the lowest ratio of 1.1:1 reported in a health promotion SROI study [Table 4]. However, because of the heterogeneity in the

**Table 4:** Range of SROI ratios by Public Health area

Public health area	Minimum SROI ratio	Maximum SROI ratio
Child Health	1.85	65.00
Environmental Health^a^	26.00	26.00
Health Care Management	1.98	7.00
Health Education^a^	7.25	7.25
Health Promotion	1.10	11.00
Mental Health	1.57	11.91
Nutrition	2.05	5.28
Sexual Reproductive Health	1.73	21.20

^a^Only one included study

350 manner of conduct of the SROI studies and indeed the economic theory that underpins the SROI methodology itself, it is not appropriate to compare the ratios to identify the most impactful or the intervention with the most value-for-money.

### Lessons learnt from previous application of SROI in public health

Five key themes emerged that captured lessons learnt from previous SROI application in public health. These are: 1) use of multiple sources of data improves trustworthiness, 2) Purchasing Power Parity (PPP) equivalents improve cost comparability, 3) beneficiaries’ ability to provide a realistic description and valuation of outcomes, 4) estimating the counterfactual should be objectively done and 5) improved transparency required throughout the SROI process.

#### Multiple sources of data improves trustworthiness

It is clear that data required for SROI studies is scarce and both the type and amount of data required are not routinely collected. One paper suggested that this is the reason why most SROI studies in developing countries depended on stakeholder consultations to generate values to be used to estimate the SROI ratio [36].

*“… so for measurement, dependence was mostly on consultation. This can be further triangulated with other data sources available internationally”* [36].

Generally, practitioners encourage organisations to gather and keep accurate data, by embedding robust and rigorous monitoring and evaluation frameworks to assess effect of interventions [64]. Where these monitoring and evaluation data are not available, then there is a need to obtain primary data. To improve confidence, accuracy and reliability [68], some authors have triangulated data obtained during a SROI study with existing secondary data [31, 35, 49, 59] or collected two or three different types of related primary data [36, 54, 61]. In cases where there is only one type of secondary data, data can be triangulated with other types of secondary data, such as was done by Bhaumik et al., who used claims from insurance providers to verify patients’ hospital visitations in a community-based care management programme for paediatric asthma [31].

#### Purchasing power parity equivalents improves cost comparability

Cost and outcomes are financially valued in SROI [8]. However, the value of a “basket of goods” bought with $1 may differ from the value of the same “basket of goods” bought with the exchange rate value of $1 in another currency.

*“Applying PPP is important in order to ensure that we do not over value or undervalue goods in different economies by using a day-to-day exchange rate. After all, the US$ will buy significantly more in Zambia than the Zambian Kwacha, which could skew the findings of the SROI evaluation”* [19].

The use of the Purchasing Power Parity (PPP), which allows for comparability across interventions and across settings [69], is proposed for valuation of both costs and outcomes in future SROI studies [19].

#### Beneficiaries’ ability to provide a realistic description and valuation of outcomes

SROI attempts to describe outcomes as perceived by a range of stakeholders, however, it has been suggested that “true beneficiaries” are better placed, compared to other stakeholder groups, to determine the outcomes accrued as a result of the intervention. In general, beneficiaries will have experienced the outcome of the intervention (or lack of the outcome) and can therefore be expected to provide more realistic valuation of the effect of the intervention (on them) than stakeholders who fund, support or implement the intervention [66].

*“Whilst by no means a perfect science, it is important to note that all monetary values, or financial proxies used to represent a programme outcome should be informed by programme beneficiaries”* [51]*.*

One study went on to sub-classify the beneficiary group into first and second tier beneficiaries depending on their proximity to the primary outcome of the intervention [45].

However, it is generally agreed that stakeholders other than the “true beneficiaries” (such as: implementers, promoters and funders) remain highly relevant with regard to the identification of these outcomes and effects that may be expected to occur following the intervention as well as in identifying other potential stakeholders and possibly making recommendations on how to improve the programme based on expert opinion. In addition, their engagement and participation through reflexive consultative processes [57] is essential to ensure that they clearly understand the needs and perspectives of the beneficiaries, for whom the intervention is intended. These are considered gains for organisations keen on making impact in the community [35, 54].

“… *the opportunity to reflect upon the history and anticipated events that were avoided was beneficial and enlightening to the group*” [35].

To make the process by which beneficiaries assign value more robust, especially with regard to financial valuation of effects (or lack of effect) of an intervention, Smith suggested that the financial proxies described by beneficiaries (which represent the value they place on the outcome in question), should be tested through further research for appropriateness and relevance. This could be achieved by integrating a proxy verification process into existing routine monitoring and evaluation procedures to ensure that proxy databases are up to date and reflect current trends and perceptions of beneficiaries [66].

#### Estimating the counterfactual should be objectively done

Authors highlight the difficulty in ascertaining what would have happened in the absence of the intervention; that is evidence of what is referred to as ‘counterfactual’ [36]. The challenges reported include the need for exhaustive data collection, both at baseline and follow-up as well increased the cost and personnel required to do this [7].

Some studies have therefore resorted to using subjective assessments to demonstrate the counterfactual. For example, a study used arbitrary percentage attribution figures by assuming that attribution is 100 % if the outcome is completely a result of the intervention and no other intervention contributed or 75 % if other interventions had some minor role to play in generating the outcome or 50 % if contribution was deemed equal from two different interventions, including the one of interest and so on [65].

*“The issue of how much of the achieved outcome can be attributable to the programme is difficult to determine with any level of objectivity, in the absence of a counterfactual, which is the norm for NGO implemented programmes of this nature”* [36]*.*

However, an estimate of the counterfactual is needed in order to be able to establish attribution (what portion of the outcome is specifically due to the intervention) and this needs to be done in an objective manner, either by using a before-and-after method or comparing the intervention group with a control group [41]. Alternatively, mapping out the underlying theory of change at the design stage of the intervention, which shows the hypothesised linkage(s) from input to impact of any intervention will go a long way in aiding establishment of the counterfactual, as this helps to clearly identify specific and relevant data required for input, output and outcomes [19, 34].

#### Improved transparency required throughout the SROI process

The most recent guide to conduct SROI includes being transparent as one of the principles for SROI [8], though a definition for the concept of “transparency” was not given. However, borrowing from mainstream research, transparency is “the benchmark for writing up research and the presentation and dissemination of findings; that is, the need to be explicit, clear, and open about the methods and procedures used” [70]. The guide states that being transparent would require SROI researchers to “demonstrate the basis on which the analysis may be considered accurate and honest, and show that it will be reported to and discussed with stakeholders” [8].

Practitioners have suggested other concrete methods to improve transparency of SROI studies. Pank and RM Insight suggested that an audit trail should be maintained throughout the study [38, 50]. Bagley suggested that a self-assurance process that allows for review of processes and comparison against benchmarks as set out in the SROI guidelines should be in place [53]. This process should detail:

*“… how each question within the accreditation criteria has been addressed within the report and provide relevant cross-references”* [53].

There is also a suggestion to create a formal process of engaging stakeholders to verify the findings and thus increase transparency of the SROI process [30, 54]. The non-profit organisation, Christian Aid referred to this as a process of “interrogating the analysis” [34].

*“Interrogate this analysis alongside partners to identify what findings are new and what simply confirm the findings and conclusions of other studies. Reach consensus on which parts of the process/analysis were most useful and instigate a process to include these in future impact analyses”* [34]*.*

## Discussion

This systematic review has helped to map the global application of the SROI methodology in public health since its first application in 2005. It has also identified best practices and lessons learnt from previous SROI studies in public health. The application of SROI to estimate the social impact and value for money of interventions is innovative and results could be used to inform policy and practice such that the most cost-beneficial interventions are implemented to solve existing public health challenges [15, 20].

One of the key challenges in conducting this systematic review was the identification of SROI studies that have been conducted in public health. There is no dedicated indexed database for SROI studies. Most SROI studies are currently published as reports in the grey literature and do not have key words and abstracts through which they can be easily retrieved.

To date, the UK is the largest proponent and user of the SROI methodology. This is consistent with the efforts of the UK Government to stimulate accountability for wider social, economic and environmental benefits to society within the Third Sector, as earlier methodologies were more focused on cost of interventions, efficiency and economies of scale [71, 72]. The steep rise in the number of SROI studies in public health between 2005 and 2011 is consistent with findings from a previous systematic review of all SROI studies [21]. This may have been due to the fact that the Office of the Third Sector launched the Measuring Social Value project in 2008 [7]. There has been a decline in the use of SROI after 2011, probably because of the discontinuing stimulus from the government or the inherent challenges needed to conduct SROI studies including cost, time and the people- and expertise-dependent nature of the methodology [6]. However, with the coming into law of the Social Value Act on 31 January 2013, requiring people who commission public services to consider how they can also secure wider social, economic and environmental benefits, the relevance of frameworks such as SROI is again highlighted [73, 74].

Additionally, there have been renewed efforts recently to apply the methodology in areas of health such as global health [75], one health (health of people, animals and environment) [76, 77], physical health [78] and maternal health [79]. All these calls recognise that the challenges that ‘limit’ the application of the methodology are not unique to SROI itself and indeed a SROI study adds value with regards to organisational accountability and reflexivity, which other frameworks rarely offer [6].

Seven SROI studies have been successfully conducted in low and middle-income countries (LMICs) [19, 29, 34, 36, 43, 51, 66], compared to 33 published studies from high-income countries [19, 28, 30–33, 35, 37–39, 41, 42, 44–50, 52–65]. This is despite the fact that LMICs receive the highest amount of aid to fund public health interventions [80] and arguably need to explore the use of robust methodologies to assess impact of such interventions. The reasons for this are not entirely clear. However, it appears from this review that paucity of reliable data may be the main reason for this [36, 81]. Triangulation, which most authors in our review suggested as a method of improving data accuracy, is a well-known method for integrating qualitative and quantitative data [82] and may potentially help to address this reliability issue. Furthermore, the awareness of the potential of the methodology to account for social impact of interventions in public health is comparatively low outside the UK. The UK, Australia and Canada are the only countries currently with a designated national SROI Network, with the membership base comprising of anyone with specific interest in the methodology [83]. Recently, the SROI Network officially confirmed its merger with the Social Impact Analysts Association (SIAA) to form Social Value International [84]. This could potentially increase global awareness amongst practitioners and researchers.

Even within the UK, evidence suggests that SROI studies are more frequently conducted within the non-profit sector and there has not been significant application of the methodology amongst academia, possibly reflected by the minimal number of SROI studies published in peer-reviewed journals. The SROI methodology evidently emerged from praxis rather than research, therefore, for the methodology to gain wider academic acceptance, its processes have to be self-reflexive, the questions being asked have to be clear and well defined, the methodology replicable and results valid [21, 85]. The rigour required to test and re-test research methodology is well developed in academia, which is why academic inputs would be key for future developments of the SROI methodology.

Despite generic guidance from the SROI Network [8, 86], this review has shown that there are differing opinions on how best to apply the SROI methodology. Firstly, there is a need to explore more scientific methods used to account for what would have happened with and without the intervention. At present, most SROI studies use subjective means, such as stakeholder consultation, to identify and value this. However, some studies have used a before and after method [35–37, 39–42], while others compared the intervention group with a control group [19, 28–34, 54]. Both of these methods are more objective and could potentially increase the reliability and validity of SROI results. Clearly, there are situations when neither of these ‘objective means’ is possible, either for practical or ethical reasons. For such cases, there is a need to provide clear guidance on how the effect was valued and how the counterfactual was determined.

This review also shows that there is no consensus regarding which stakeholders should be included to account for the outcomes of the intervention(s) assessed. Some authors have included only stakeholders who directly benefitted from the intervention and not all stakeholder groups. Those who experienced the outcomes should be asked to value the benefits (or lack of) themselves; as this may potentially be a closer to true reflection of the real impact of public health interventions. Other stakeholders (implementers, funders or promoters) are not as well placed to describe experiences of beneficiaries. The proposition here is, if an “investment” has been earmarked for the benefit of a group of people, then the “return on investment” should be for what the investment has done for those people. Inclusion of outcomes from other stakeholder groups may lead to overestimation of the social value of the investment, which is not in line with the principles of the SROI methodology [8]. In addition, previous impact evaluation methodologies such as the cost-utility and cost-benefit analysis focus only on the beneficiaries [87].

For this review, we used Krlev et al.’s 12-point quality assessment framework. This framework was selected because it is the first and only publicly available framework for judging quality of SROI reports. Secondly, the framework incorporates critical and sound research insights, such as how SROI studies account for what would have happened without the intervention. We confirmed the fitness for purpose of the Krlev et al. framework by using a tool developed by Gough [23]. We also shared the framework with a SROI practitioner and an impact evaluation practitioner for their expert opinion, who both recommended it for use.

There is clearly a need for SROI practitioners and public health researchers to collaborate in developing a more widely acceptable and perhaps more robust quality assessment framework for public health SROI studies, similar to the Consolidated Health Economic Evaluation Reporting Standard (CHEERS) framework for economic evaluations [88]. This is even more pertinent as the authors of the quality assessment framework used in this review recognised the limitations [21].

From the findings of this review, it appears that the quality of public health SROI studies has not significantly improved over the years. Some of the more recent studies did not conduct sensitivity analysis and/or did not account for the counterfactual scenario objectively [31, 33, 46, 47, 52, 57, 65]. In contrast, there is an earlier study, which has fully adhered to the SROI principles and guidance and accounted for the counterfactual scenario [36]. Quality seems to be analyst dependent rather than time dependent (as quality has not improved over time) or assurance dependent (as the paid internal peer review service only checks if the conduct has aligned to the SROI Network principles [89]). This means that skills of SROI researchers can potentially be increased through training in the methodology itself. The assurance process of the SROI Network, which is a form of peer review, is worth following, though it is considered inconsistent [90]. The process as it is today could adopt best practices including a peer review process such as is used in research proposal and scholarly publication (including that peer review is free and uses people of similar competence to evaluate work of others) [91].

The Roberts Enterprise Development Fund (REDF) described current approaches to SROI as lacking the systemization and links to established information systems that can ensure basic levels of reproducible data, data integrity, and comparability. In a call to action, the REDF proposed that the “next generation SROI” should make use of credible financial and social outcomes systems for collection of costs and outcomes data. The organisation also proposed that these systems should be linked so as to increase comparability of results and ensure that only meaningful and reliable results will be generated. This would ultimately lead to wider use of the model [92]. In this regard, to account for cost variations and currency exchange rate differences, best practices such as the use of Purchasing Power Parity (PPP) value of monies will improve the comparability of results, at least for similar interventions. In addition, installing a framework to support conduct of SROI in programmes at baseline such as was done by Bhaumik et al. who tracked the number of children visiting emergency department and costs throughout the community asthma initiative from one year before intervention to three years after [31], may help to build more reliable outcomes databases. Only high quality data can yield the robust values required to account for value for money.

This review needs to be interpreted bearing in mind the following limitations. Firstly, only published public health focused SROI studies were included in the review. There are probably unpublished SROI studies not in the public domain. While this is a recognised limitation, attempts were made to request print versions of SROI studies from any authors that the reviewers became aware of during the conduct of the review.

Furthermore, the limited number of SROI studies published per public health area and the heterogeneity in conduct of the studies limited the capacity to aggregate findings from related SROI studies. Finally, the quality of the SROI studies is a limitation. Overall assessment of the quality of SROI studies included in this review provided an above average score. However, a sub-analysis revealed key weaknesses in choice of design for accounting for outcomes. While this does not mean that the SROI studies themselves do not carry valuable information, the conduct of the research could have been better.

There is a need to establish a comprehensive database to index SROI studies to allow for easier retrieval. Practitioners need to engage with academics to develop the methodology further and clear guidance is needed to systematise the procedures for applying the SROI methodology.

## Conclusions

The international development community continues to invest significantly in public health. A culture of accountability and “value for money” is central to monitoring and evaluation of public health projects, programmes and policies. In times of austerity, robust and innovative tools are needed. The SROI methodology provides a platform to systematically account for broader outcomes of interventions and the value for money of such interventions. SROI is very relevant and applicable, especially as the global focus shifts from “output” to “impact” and from “generous giving” to “accountable giving” [94]. It aids identification of the most impactful, cost-beneficial and culturally sensitive public health interventions. It is however clear that the methodology will benefit from increased synergy between SROI practitioners and public health researchers in order to be able to account for the real and broad impact of interventions more robustly.

## Additional files

Additional file 1:
**PRISMA checklist.**
Additional file 2:
**Quality assessment of public health SROI studies.**
Additional file 3:
**Summary table on application of SROI on public health interventions.**

## Abbreviations

CBA: Cost-benefit analysis
CEA: Cost-effectiveness analysis
CUA: Cost-utility analysis
CHEERS: Consolidated health economic evaluation reporting standard
LMIC: Low and middle income countries
nef: New economics foundation
NGO: Non-Governmental Organisation
PPP: Purchasing power parity
PRISMA: Preferred reporting items for systematic reviews and meta-analyses
REDF: Roberts enterprise development fund
SIAA: Social impact analysts association
SROI: Social return on investment
SROIN: Social return on investment network
UK: United Kingdom
VfM: Value for money
